# Supplementary material for: Genomic Surveillance of SARS-CoV-2 in Healthcare Workers: A Critical Sentinel Group for Monitoring the SARS-CoV-2 Variant Shift
Source: Viruses. 2023 Apr 17;15(4):984. doi: 10.3390/v15040984 (PMC10146896; doi:10.3390/v15040984)
Supplement: Supplementary file 1 [file viruses-15-00984-s001.zip › viruses-2341144-supplementary.pdf]

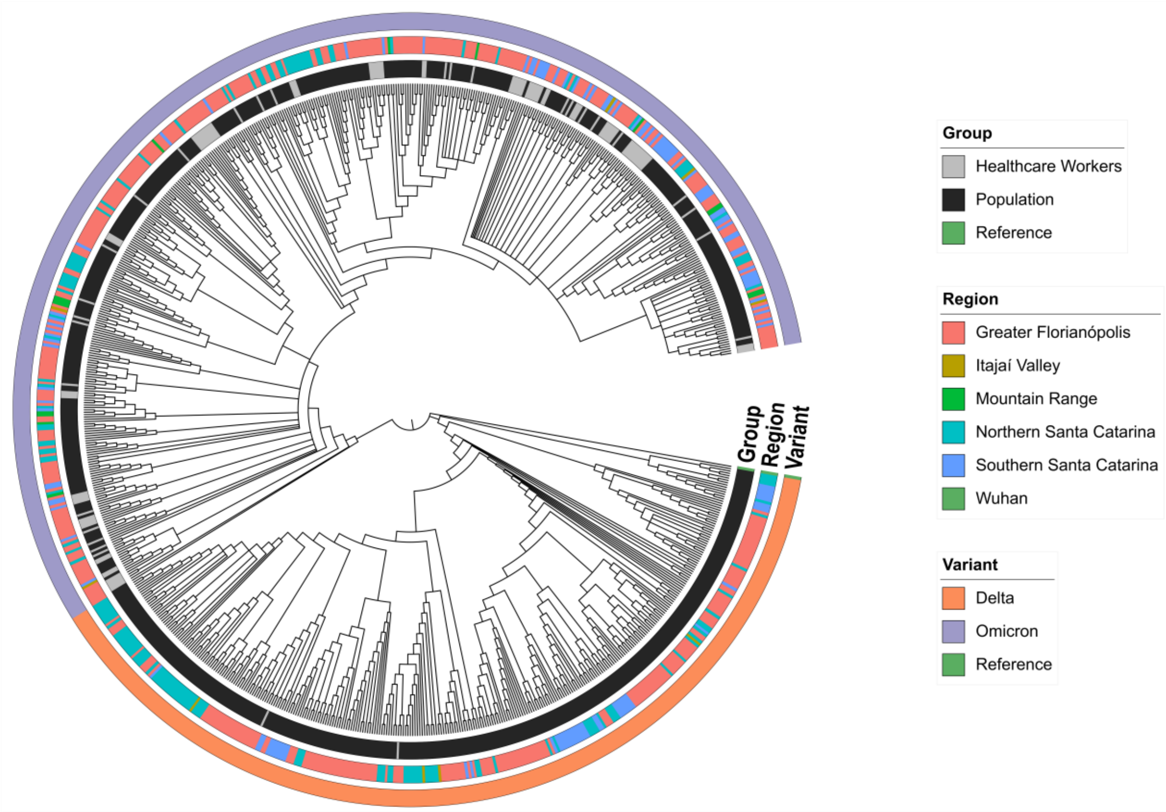

Figure S1: Phylogenetic reconstruction of SARS-CoV-2 sequences distinguished between general population and healthcare workers. Samples correspond to the period between 17 November 2021 and 21 February 2022. The phylogenetic tree was reconstructed with 1000 iterations for Ultra-Fast Bootstrap and SH-like approximate likelihood ratio test (SH-aLRT). Reference (SARS-CoV-2 reference genome-NC_045512).
